# *Bifidobacterium breve* intervention combined with environmental enrichment alleviates cognitive impairment by regulating the gut microbiota and microbial metabolites in Alzheimer’s disease mice

**DOI:** 10.3389/fimmu.2022.1013664

**Published:** 2022-09-20

**Authors:** Guangsu Zhu, Min Guo, Jianxin Zhao, Hao Zhang, Gang Wang, Wei Chen

**Affiliations:** ^1^ State Key Laboratory of Food Science and Technology, Jiangnan University, Wuxi, China; ^2^ School of Food Science and Technology, Jiangnan University, Wuxi, China; ^3^ (Yangzhou) Institute of Food Biotechnology, Jiangnan University, Yangzhou, China; ^4^ National Engineering Center of Functional Food, Jiangnan University, Wuxi, China

**Keywords:** Alzheimer’s disease, *Bifidobacterium breve*, environmental enrichment, gut microbiota, acetate, tryptophan metabolism, gut–brain axis

## Abstract

Alzheimer’s disease (AD) is characterized by behavioral and cognitive impairments and its increasing prevalence imposes a healthcare burden on society. To date, most intervention studies have only focused on a single AD-related factor and have yielded modest cognitive improvements. Here, we show that environmental enrichment (EE) training combined with *Bifidobacterium breve* CCFM1025 intervention significantly alleviated amyloid-β (Aβ)-induced cognitive impairment and inhibited neuroinflammation in mice. Moreover, we found that EE combined with *B. breve* CCFM1025 treatment restored AD-associated gut microbiota dysbiosis and reversed microbial metabolites changes. By integrating behavioral and neurological data with metabolomic profiles, we corroborated the microbiota–metabolite–brain interactions, with acetate and tryptophan metabolism as potential drivers. Taken together, our results provide a promising multidomain intervention strategy to prevent cognitive decline and delay the progression of AD through a combination of dietary microbiome-based approaches and lifestyle interventions.

## Introduction

Alzheimer’s disease (AD) is a neurodegenerative disease characterized by the deposition of amyloid plaques, the formation of neurofibrillary tangles, cognitive impairment, and neuroinflammation (1). Accumulating evidence from both animal models and human cohorts indicate that AD is related with gut microbiota dysbiosis, including quantitative and qualitative changes in bacterial diversity and microbiome composition (2). Among the differentially abundant bacteria, salutogenic taxa, such as *Bifidobacterium* and *Turicibacter*, are decreased in abundance, whereas pathogenic taxa, such as *Bilophila* and *Gemella*, are significantly increased in patients with AD compared with healthy individuals (3). Further research has demonstrated that the clinical indicators of AD are negatively correlated with the abundance of lactate- and propionate-producing bacteria, such as *Akkermansia* and *Bifidobacterium* (4). Indeed, as the gut microbiota has been shown to bidirectionally communicate with the brain, the gut–brain axis has been proposed (5). Moreover, mounting mechanistic evidence suggests that the gut microbiome impacts brain function through microbiota–gut–brain connections, which may be regulated by neuronal and immune-mediated signaling (6). At the intersection of neuroscience and microbiology, the gut microbiome is a dynamic entity that changes in composition and structure throughout the host’s lifespan. Thus, the modulation of the intestinal microbiota through dietary intervention offers a novel strategy to delay the progression of AD.

As determined by metabolomic screening, the gut microbiome generates various small molecule metabolites to communicate with the host (7). Broadly, these microbial metabolites may be categorized into three types: 1) dietary metabolites unique to bacteria, including short-chain fatty acids (SCFAs) and amino acids; 2) bacteria-modified bile acids; and 3) host–bacteria co-metabolites, such as neurotransmitters (7). A large proportion of microbiota-derived metabolites are able to cross the blood–brain barrier (BBB) and thus enter the brain and affect brain function and host health (8). Recent technological advancements in metabolomic analyses have made it possible to detect microbiota-derived metabolites. Moreover, the development of multi-omics techniques has pushed the limits of the delineation of their regulatory mechanisms. Specific metabolites closely linked with cognition have been observed in the hippocampus, indicating that these metabolites may serve as active drivers of the microbiota–gut–brain interaction (9). Despite increased awareness of the potential function of the intestinal microbiota and microbial metabolites in AD, mechanistic links underlying the gut–brain interaction remain to be elucidated.

Lifestyle interventions to promote brain health, including diet, exercise, and social and intellectual stimulation strategies, can improve cognition in older individuals with AD (10). However, most previous intervention studies have only targeted a single factor and have yielded modest cognition improvements. Given the multifactorial aetiology of AD, multidomain interventions that target several risk factors and mechanisms simultaneously may be critical for an optimal preventive effect (10). Psychobiotics, which produce and deliver neuroactive substances, are a class of probiotics that benefit patients with psychiatric illnesses (11). As a major modulator of gut microbiota dysbiosis and brain function, the central role of selective psychobiotics in neurodegenerative diseases, through interactions with the gut–brain axis, is gaining broader acceptance (12). Environmental enrichment (EE) refers to the use of housing conditions that facilitate enhanced sensory, cognitive, motor, and social stimulation (13). Typically, EE for animals involves housing them in relatively spacious cages containing objects of different types and shapes (14). As a combination of cognitive training and physical exercise, EE has numerous protective effects on the brain, such as promoting adult hippocampal neurogenesis, improving memory and learning, and enhancing synaptic plasticity (13). Moreover, exposing mice to EE changed the gut microbiota and microbial metabolites; further, the transfer of fecal microbiota from EE-mice enhanced brain plasticity in standard environment mice (15). Thus, EE is considered to be a promising strategy to improve brain function and lower the risk of AD (16). However, few studies have assessed whether EE training combined with probiotic intervention ameliorates AD-associated pathology and behavioral symptoms in mice, and the underlying mechanisms remain poorly understood.

*Bifidobacterium breve* CCFM1025 is a promising psychobiotic strain that has been isolated from fecal samples of healthy humans (17, 18). It has been shown to have several neuroprotective effects against AD and depression, and the underlying mechanisms involve the suppression of neuroinflammation, modulation of the microbiota, and the ability to colonize the gastrointestinal tract (19, 20). The aim of this study was to investigate the effects of combined *B. breve* CCFM1025 with EE treatment on cognition and the gut microbiome and its metabolites in mice with AD. To this end, we established a mouse model of AD and evaluated the changes in behavioral deficits, neuroinflammation, and the diversity and composition of the gut microbiota after the exposure of AD mice to EE in conjunction with *B. breve* CCFM1025 intervention. We also identified significantly altered metabolome signatures and further assessed the role of microbial metabolites in regulating AD. Here, we report that combined EE and *B. breve* CCFM1025 significantly improves cognitive function and inhibits neuroinflammation by restructuring the gut microbiome and regulating acetate and tryptophan metabolism.

## Materials and methods

### Bacterial treatment

*B. breve* CCFM1025 was cultured under anaerobic conditions at 37°C in modified De Man, Rogosa, and Sharpe broth supplemented with 0.05% (w/v) L-cysteine. Freshly cultured bacterial cells were collected by centrifugation (8,000 × g for 10 minutes at 4°C), and then washed and resuspended with 30% (v/v) glycerol. Aliquot resuspended bacteria cells in sterilized tubes and store at -80°C until use. The gradient dilution method was used to determine the number of bacteria cells. For oral administration, each aliquoted bacteria sample was washed twice and resuspended in phosphate-buffered saline (PBS) to reach a concentration of surviving bacteria at 1 × 10^9^ colony-forming units/mL.

### Animal experiments

Male adult C57BL/6J mice (8 weeks old) were purchased from the Model Animal Research Center of Nanjing University (Nanjing, China) and maintained at the Animal Center of Jiangnan University as previously described (17). The experimental procedures were approved by the Animal Experimentation Ethics Committee of Jiangnan University (JN. No20200630c0960909[123]).

**Figure 1:**
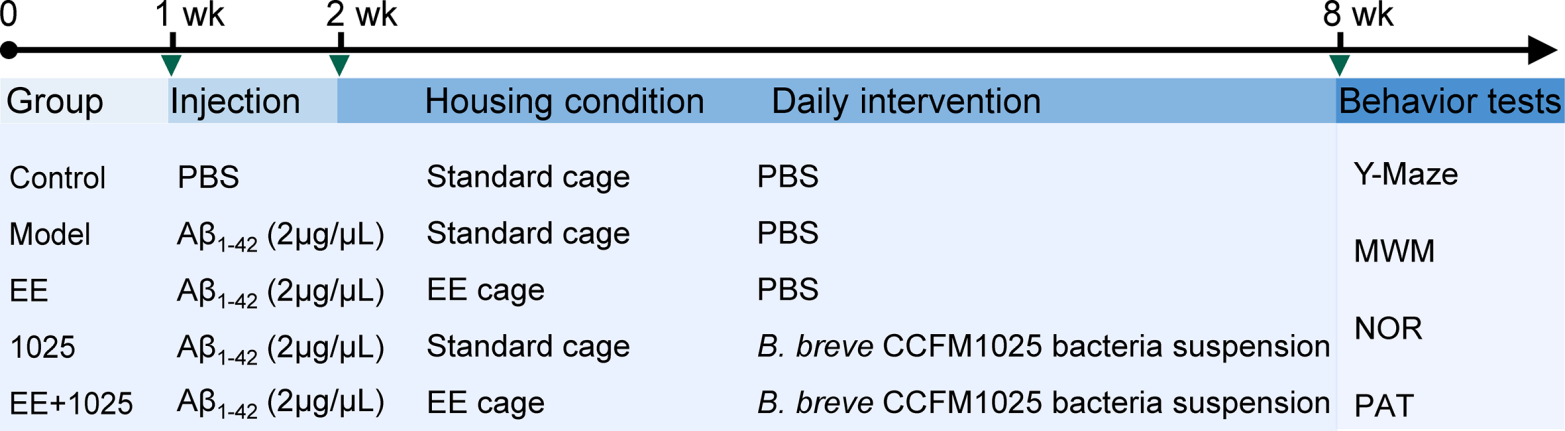
Schematic overview of the experimental design and study timeline. Vertical bars represent 1 week. wk, week; EE, Environmental enrichment; Aβ, Amyloid-β; PBS, phosphate-buffered saline; MWM, Morris water maze; NOR, novel object recognition; PAT, passive avoidance test.

After acclimatization, the mice were divided into five groups. The AD model was established by intrahippocampal injection of an amyloid beta (Aβ_1-42_) oligomer as previously described (17). The volume of intrahippocampal Aβ_1-42_ injection was 1 μL at a concentration of 2 μg/μL, whereas mice in Control group received equal volume of PBS. The mice were either housed in standard laboratory cages without any stimulation or in an EE cage (4–5 mice per cage). The mice were administered 200 μL of bacterial suspension or sterile PBS as a vehicle control by gavage once a day for 6 weeks. The details of the treatment protocol for each group and the experimental timeline are shown in **Figure 1**.

### Environmental enrichment

For EE, the mice were kept in large cages (2.893 × 104 cm^3^) and provided with running wheels, a red transparent mouse house, colored plastic tubes, wooden blocks, pressed cotton squares, and objects of different shapes (14). All enrichment items were disinfected or sterilized. Some of the items in the EE cages were replaced once a week, but the running wheel was kept for the entire experimental period. The shape and arrangement of some of the items were changed weekly to increase novelty.

### Behavioral tests

Behavioral tests were performed in a blinded manner to measure different types of learning and memory, and mice were allowed to rest between tests. As previously described (9, 17), we performed the following four behavioral tests: 1) a Y-maze to evaluate spontaneous and short-term working memory, 2) a Morris water maze (MWM) to assess spatial learning and memory, 3) a novel object recognition task to measure short-term recognition memory, and 4) a passive avoidance test to assess retention memory. The tests were conducted under dimmed lighting. The detailed protocol for behavioral tests is described in the Supplementary methods. All behavioral tests were recorded and analyzed using Ethovision 11.5 (Noldus, Wageningen, the Netherlands).

### Fecal and tissue sample collection

After the completion of behavioral tests, the mice were placed in an empty cage until defecation. Fecal samples were immediately collected, snap-frozen, and stored at -80°C for subsequent microbiome and metabolome sequencing. The mice were then deeply anaesthetized and decapitated. Brains were collected and hippocampal tissues were dissected. Aliquoted hippocampal tissues were stored at -80°C for subsequent analyses.

### Enzyme-linked immunosorbent assays

Hippocampal tissues were homogenized in pre-cooled PBS supplemented with protease inhibitor. The samples were centrifuged at 5,000 × g for 10 minutes at 4°C to collect supernatants. The supernatants were then used to measure Aβ_1-42_, brain-derived neurotrophic factor (BDNF), and interleukin 6 (IL-6) levels. For the analyses of synaptophysin (SYP), fibronectin type III domain-containing protein 5 (FNDC5), and transforming growth factor-β1 (TGF-β1), the supernatants were further diluted in cold PBS. All of these protein levels were measured using enzyme-linked immunosorbent assay kits from Elabscience (Wuhan, Hubei, China) according to the manufacturer’s protocol.

### Fecal 16S rRNA sequencing and bioinformatic analysis

Microbial genomic DNA was extracted from fecal samples using a FastDNA Spin Kit (MP Biomedical, Irvine, CA, USA). The V3-V4 region of bacterial 16S rRNA was then amplified using a universal primer pair (341F and 806R). The differential detection of *Bifidobacterium* species was performed using primers designed to target the *groEL* gene. PCR products were purified and quantified using a Qubit dsDNA Assay Kit (Life Technologies, Invitrogen, Carlsbad, CA, USA). Purified amplicons were pooled and paired-end sequencing was performed on a MiSeq PE300 platform (Illumina, San Diego, CA, USA).

Raw data were processed using the QIIME tools version 2.0 and bioinformatic analyses of the microbiota were performed. α-Diversity was calculated based on species richness and evenness using Chao 1, Shannon, and Simpson indices. β-Diversity was assessed based on the Bray–Curtis distance and was visualized *via* principal coordinate analysis (PCoA). The differential abundance of microbial taxa was calculated using linear discriminant analysis effect size (LEfSe) and visualized using a taxonomic cladogram tree. Metagenomes of the gut microbiome were computed from 16S rRNA sequences based on Phylogenetic Investigation of Communities by Reconstruction of Unobserved States (PICRUSt), and functional pathways were predicted using the Kyoto Encyclopedia of Genes and Genomes (KEGG) orthology.

### Metabolomics

For fecal metabolomic analyses, metabolites were extracted as detailed in Mars et al. (21). The metabolite samples were analyzed with an UItiMate 3000 high performance liquid–chromatography (HPLC) system (Thermo Fisher Scientific, MA, USA) coupled to a high-resolution Q Exactive mass spectrometer (Thermo Fisher Scientific, MA, USA). The detailed protocol for fecal metabolomics analysis, including metabolite sample preparation and the HPLC-MS analysis parameters are described in the Supplementary methods. The metabolomic data analysis were detailed in a previous report (19).

Fecal SCFAs, including acetate, propionate and butyrate, were extracted and quantified as previously described (22). For metabolites extraction, 30 mg fecal samples were weighed and soaked in saturated sodium chloride for 30 min. After homogenizing, 20 μL of H_2_SO_4_ (10% (v/v)) were added to each sample for acidification. Then, 800 μL of diethyl ether were added and mixed thoroughly. The supernatants were analyzed on gas chromatography–mass spectrometry (Thermo Fisher Scientific, MA, USA) with a Rtx-Wax column (column length: 30 m, inner diameter: 25 µm).

### Statistical analysis

Statistical analysis was performed using Prism version 8.0.2 (GraphPad, San Diego, CA, USA) and SPSS version 22 (IBM, Armonk, NY, USA). Data are presented as mean values ± standard error of the mean or the median ± interquartile range. Before further statistical analyses, data were tested for normal distribution using the Shapiro–Wilk test and Q-Q plots. A one-way analysis of variance (ANOVA) with Holm–Sidak test or Student’s t-test was used for the parametric analysis of differences between groups and a Kruskal–Wallis test followed by Dunn’s test or Welch’s t-test was performed for nonparametric data variance analysis. Values of *P* < 0.05 were considered to be statistically significant. Details of the statistical tests, the number of samples, and significance cut-offs are described in each figure legend. The network correlation between variables was determined using Spearman’s correlation coefficients and visualized with Cytoscape version 3.8.2 (Institute for Systems Biology, Seattle, WA, USA).

## Results

### EE + *B. breve* CCFM 1025 treatment alleviates AD-induced cognitive and behavioral deficits

**Figure 2:**
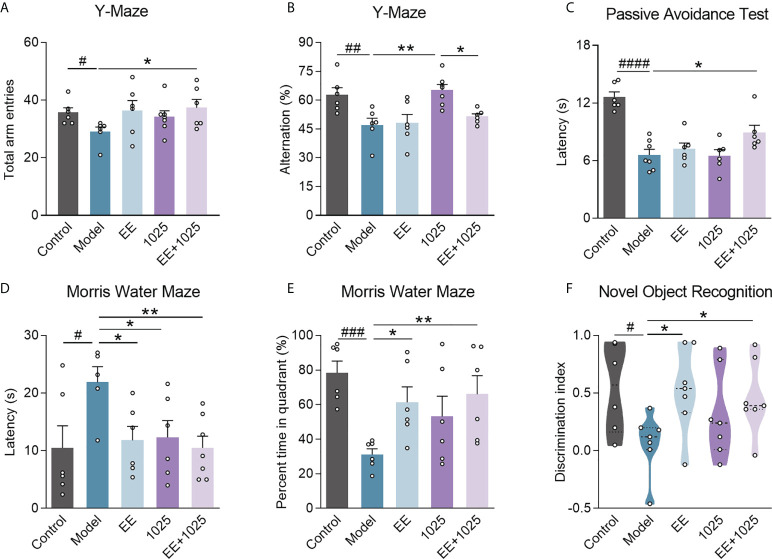
EE combined with *B*. *breve* CCFM1025 treatment alleviates AD-induced cognitive and behavioral deficits. Total arm entries **(A)** and spontaneous alternation behavior **(B)** in Y-maze test (n = 6-8). **(C)** Escape latency of passive avoidance test (n = 6-8). The escape latency **(D)** and percent time in target quadrant **(E)** during the probe phase (day 6) of the Morris water maze. **(F)** Discrimination index in the novel object recognition task (n = 6-8). Black horizontal dashed lines in violin plots depict medians, the bottom and top lines represent, respectively, the 25th and 75th percentile. Data are presented as mean ± standard error of the mean (mean ± SEM). Each data point represents one independent mouse. Control vs. Model: ^#^ *P* < 0.05, ^##^ *P* < 0.01, ^###^ *P* < 0.001, ^####^ *P* < 0.0001 by unpaired student’s t-test; *P < 0.05, **P < 0.01 as determined by one-way ANOVA.

To explore the ameliorative effects of *B. breve* CCFM1025 combined with EE on AD-associated cognitive impairment, we performed behavioral tests in AD mice treated with or without either *B. breve* CCFM1025 or EE for 6 weeks after intersection hippocampal injection of Aβ_1-42_ (**Figure 1**). In Y-maze tests, the total numbers of arm entries and spontaneous alternation behaviors were significantly lower in the Aβ_1-42_-treated group than the sham treatment group (**Figures 2A, B**). Mice treated with EE or CCFM1025 alone showed a slight, but not statistically significant, increase in the total number of arm entries (**Figure 2A**). Mice treated with EE alone failed to show improvement in alternation behavior, whereas those treated with *B. breve* CCFM1025 alone did show an improvement (**Figure 2B**). In contrast, mice treated with EE + *B. breve* CCFM1025 showed improved working memory in the Y-maze (**Figures 2A, B**). Similarly, in a passive avoidance test, EE or *B. breve* CCFM1025 treatment alone resulted in no observed improvements in latency time; however, mice treated with EE + *B. breve* CCFM1025 showed a markedly prolonged latency time compared with model mice (**Figure 2C**).

In the retention memory task of the MWM probe test, on day 6, all three groups receiving EE and/or *B. breve* CCFM1025 showed significantly shortened escape latency compared with model mice, whereas EE + *B. breve* CCFM1025 mice performed better (**Figure 2D**). Additionally, of all the groups tested, untreated AD mice spent the shortest amount of time in the targeted quadrant, whereas the time spent in the targeted quadrant was significantly extended for mice treated with EE + *B. breve* CCFM1025 (**Figure 2E**). In the novel object recognition task, AD mice exposed to EE, with or without *B. breve* CCFM1025 treatment, showed similar increases in discrimination ratio (**Figure 2F**).

### EE + *B. breve* CCFM1025 treatment inhibits AD-related neuroinflammation and synaptic impairments in the brain

**Figure 3:**
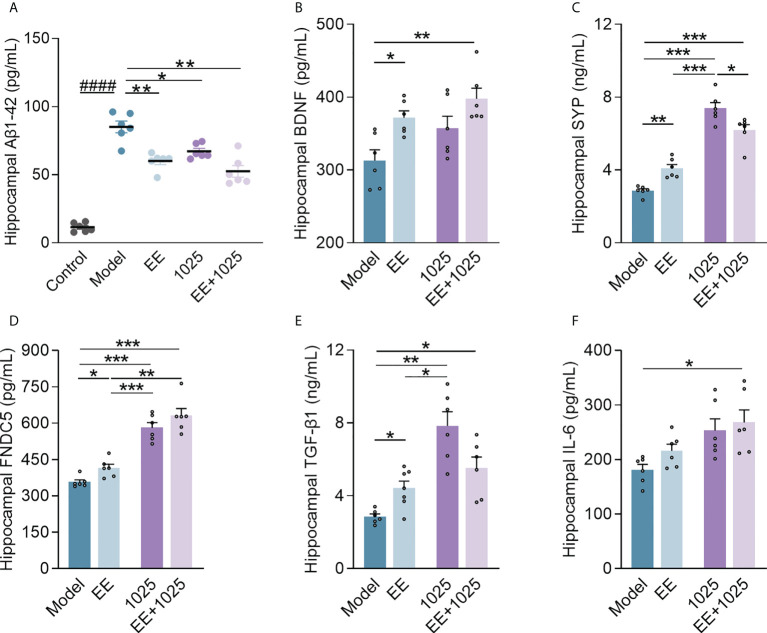
EE combined with *B*. *breve* CCFM1025 treatment inhibits AD-related neuroinflammation and synaptic impairments in the brain. **(A)** Hippocampal Aβ_1-42_ levels (n = 6–8). **(B)** Hippocampal BDNF levels (n = 6–8). **(C)** Hippocampal SYP levels (n = 6–8). **(D)** Hippocampal FNDC5 levels (n = 6–8). **(E)** Hippocampal TGF-β1 levels (n = 6–8). **(F)** Hippocampal IL-6 levels (n = 6–8). Data are presented as mean ± standard error of the mean (mean ± SEM). Each data point represents one independent mouse. Control vs. Model: ^####^ *P* < 0.0001 by unpaired student’s t-test; *P < 0.05, **P < 0.01, ***P < 0.001 as determined by one-way ANOVA.

To compare the extent of brain damage in AD mice, we examined amyloid deposition in brain samples from each group. Significant accumulation of hippocampal Aβ_1-42_ was seen in Aβ-injected model mice compared with sham-treated control mice (**Figure 3A**). Notably, oral *B. breve* CCFM1025 treatment decreased hippocampal Aβ_1-42_ levels. Moreover, the accumulation of hippocampal Aβ_1-42_ was significantly decreased in the EE-treated groups, with the EE + *B. breve* CCFM1025 group showing the greatest effect.

Next, to confirm whether EE and *B. breve* CCFM1025 treatments enhance synaptic plasticity, we measured the levels of BDNF, SYP, and FNDC5 in the hippocampal homogenates of Aβ-injected mice. We observed a highly significant increase in all three synaptic proteins in mice treated with EE compared with those housed in a standard cage (**Figures 3B–D**). Notably, mice treated with EE + *B. breve* CCFM1025 showed greater increases in the levels of hippocampal BDNF, FNDC5, and SYP than mice treated with EE only. Moreover, we found that treatment with *B. breve* CCFM1025 alone increased the levels of hippocampal SYP and FNDC5, but not the levels of BDNF. These findings indicated that the exposure of mice to EE was critical for enhancing synaptic plasticity.

To further explore the neuroprotective effect of EE combined with *B. breve* CCFM1025 on neuroinflammation, we measured cytokine concentrations in the brains of AD mice. The TGF-β1 levels increased in the hippocampal samples of mice in all three groups treated with EE and/or CCFM1025 compared with untreated AD mice housed in a standard cage (**Figure 3E**). Specifically, only treatment with EE + *B. breve* CCFM1025 significantly increased IL-6 levels in hippocampal tissues (**Figure 3F**). Given that TGF-β1 and IL-6 have been reported to regulate neurogenesis and are selectively elevated following exercise, EE-induced exercise combined with the psychobiotic potential of *B. breve* CCFM1025 may improve brain function under the pathological conditions of AD.

### EE + *B. breve* CCFM1025 treatment improves gut microbial dysbiosis in Aβ-injected mice

**Figure 4:**
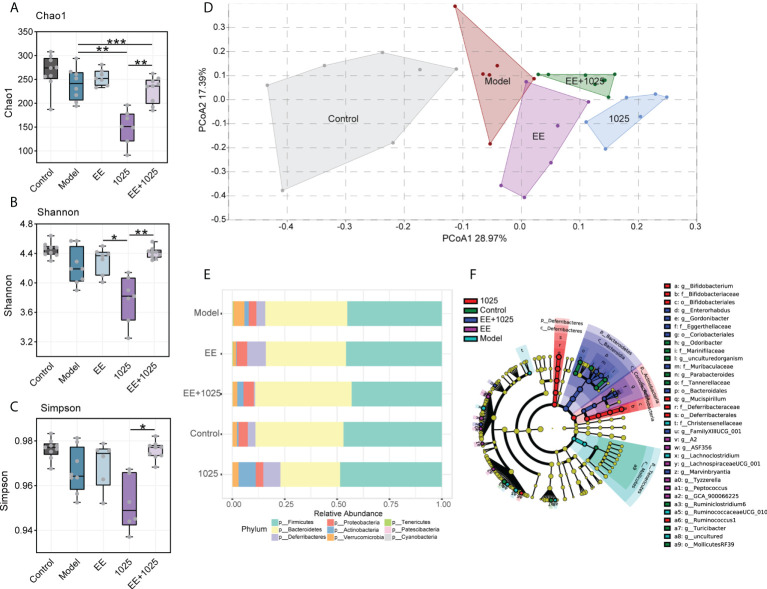
Changes in the diversity and structure of gut microbiome in different groups. **(A–C)** Microbial α-diversity, indicted by Chao 1, Shannon and Simpson indices (n = 6–8). In the box plot, the bottom and top are, respectively, the 25th and 75th percentile, a line within the box marks the median. Whiskers above and below the box indicate 1.5 interquartile range of the lower and upper quartile, respectively. Each data point represents one independent mouse. *P < 0.05, **P < 0.01, ***P < 0.001 as determined by one-way ANOVA. **(D)** Principal coordinates analysis (PCoA) based on Bray-Curtis distance (PERMANOVA, *P* = 0.0001; n = 6–8). **(E)** The relative abundance of bacteria at the phylum level (n = 6–8). **(F)** Linear discriminant analysis (LDA) effect size (LEfSe). Differential taxa are labeled with tags and annotated in the right panel. Data were computed with an LDA score above 2.00 and *P* value below 0.05 for the factorial Kruskal–Wallis test.

Compared with control mice, gut microbiota dysbiosis in Aβ-injected model mice was observed using fecal 16S rRNA gene sequencing analysis. We analyzed α-diversity using Chao 1, Shannon and Simpson indices. While model mice showed no difference compared with control mice, oral treatment with *B. breve* CCFM1025 resulted in lower diversity and richness than EE group (**Figures 4A–C**). However, these decreased α-diversity indices in CCFM1025 group were significantly restored by EE+1025 treatment.

To analyze the β-diversity of the gut microbiota, we performed PCoA based on the Bray–Curtis distance. Significant differences in gut microbiome structures and signatures were observed between the model and control groups (**Figure 4D**). Specifically, *B. breve* CCFM1025-treated mice showed a greater change in β-diversity than EE-treated mice, suggesting that *B. breve* CCFM1025 administration led to a dramatic change in the microbiome signature (**Figure 4D**).

**Figure 5:**
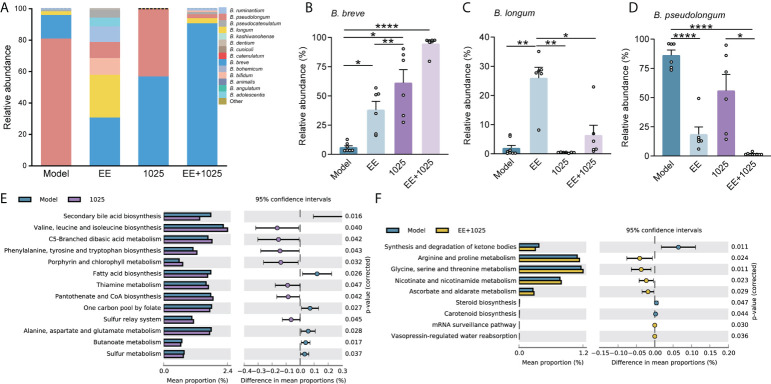
EE combined with *B. breve* CCFM1025 treatment alters the *Bifidobacterium* spp. and affects the gut microbiome on functional level. **(A)** Distribution of *Bifidobacterium* species in the *Bifidobacterium* genus (n = 6). **(B–D)** The relative abundance of *B. breve*, *B. longum* and *pseudolongum* (n = 6). Data are presented as mean ± standard error of the mean (mean ± SEM). Each data point represents one independent mouse. *P < 0.05, **P < 0.01, ****P < 0.0001 as determined by one-way ANOVA. **(E)** Differential microbial functions between model and CCFM1025 groups. **(F)** Differential microbial functions between model and EE+1025 treated groups. Statistical difference of the gene contents between the two groups is screened by Welch’s-t test with *P* < 0.05.

At the phylum level, the most abundant taxa in each group of mice were Firmicutes and Bacteroidetes. Notably, there was a greater abundance of members of the Actinobacteria phylum in *B. breve* CCFM1025-treated mice than in Aβ-injected model mice. However, in EE-treated mice, the phylum Proteobacteria was enriched (**Figure 4E**). At the genus level, probiotic bacteria, such as *Bifidobacterium*, *Mucispirillum*, and *Ruminococcus*, were markedly enriched in the *B. breve* CCFM1025 group, whereas *Enterorhabdus* and *Gordonibacter* (classified in the order Coriobacteriales) were more prevalent in the EE + *B. breve* CCFM1025 group (**Figure 4F**). We also focused specifically on abundance of species from *Bifidobacterium* in each group of mice. Notably, the relative abundance of *Bifidobacterium* spp. was significantly different in each group (**Figure 5A**), and the abundance of *B. breve* was greater in in the *B. breve* CCFM1025-treated groups (**Figure 5B**), which may be due to the colonization of *B. breve* induced by its administration by oral gavage for 6 weeks. However, the abundance of *B. longum* was markedly higher in EE treated group as compared with other groups (**Figure 5C**). Importantly, EE alone or combined with *B. breve* CCFM1025-treated mice showed a decreased abundance of *B. pseudolongum*, which was a prevalent species in model mice (**Figure 5D**).

To gain insights into the potential pathways that may influence the host metabolic output, we performed KEGG pathway analysis of gut microbiome genes. Eleven pathways were identified differential between model and *B. breve* CCFM1025 groups, among which valine, leucine and isoleucine biosynthesis, and phenylalanine, tyrosine and tryptophan biosynthesis were statistically activated by *B. breve* CCFM1025 intervention (*P* < 0.05, **Figure 5E**). In contrast, of the seven differential pathways between model and EE + *B. breve* CCFM1025 groups, tryptophan metabolism, arginine and proline metabolism, and glycine, serine and threonine metabolism were particularly upregulated in the EE + *B. breve* CCFM1025 group (*P* < 0.05, **Figure 5F**). Together, these metabolic outputs may be regulated by *B. breve* CCFM1025 alone or in combination with EE, predominantly related to amino acid metabolism.

### EE + *B. breve* CCFM1025 treatment regulates microbial metabolites in Aβ-injected mice

Based on the role of the gut microbiome in regulating brain deficits induced by AD, we hypothesized that microbial metabolites may be potential factors that affect cognition. Thus, we first performed untargeted metabolomic analyses to detect candidate microbiota-derived metabolites in fecal samples of control, model, and EE + *B. breve* CCFM1025-treated mice.

**Figure 6:**
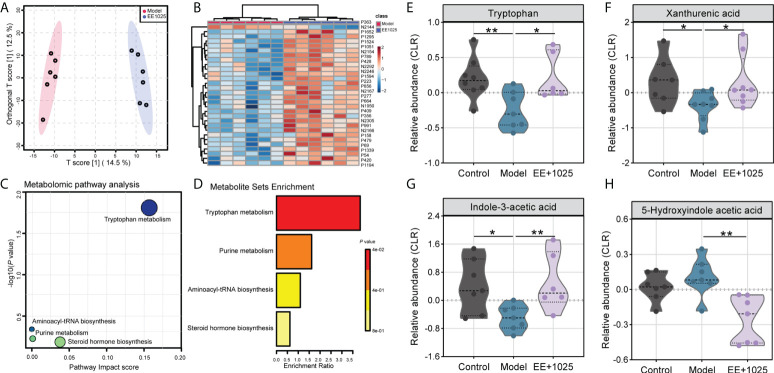
EE combined with *B. breve* CCFM1025 treatment regulates microbial metabolites in Aβ-injected model mice. **(A)** OPLS-DA score plot between model and EE + *B. breve* CCFM1025 groups. **(B)** Hierarchical cluster analysis of differential metabolites between model and EE + **(*B*)** *breve* CCFM1025 groups in fecal samples (top 30). The name of differential metabolites was listed in **Supplementary Table 2**. **(C)** Scatterplot showing results of MetaboAnalyst Pathway analysis using the Mus musculus (mouse) pathway library. **(D)** Overview of metabolite set enrichment in feces according to the KEGG database. **(E–H)** The relative abundance of tryptophan metabolites tryptophan, xanthurenic acid, indole-3-acetic acid, and 5-hydroxyindole acetic acid. The y axis shows CLR-transformed metabolite concentrations. Black horizontal dashed lines in violin plots depict medians, the bottom and top lines represent, respectively, the 25th and 75th percentile. *P < 0.05, **P < 0.01 as determined by Mann–Whitney test.

A total of 5406 metabolites (3240 with defined names) were identified in fecal samples. Aβ-injection induced significant differences in fecal metabolites, with model and control group clustering separately (**Supplementary Figure 1A**). Among all of these metabolites matched to the database, we found the abundance of 87 metabolites were significantly altered between control, model, and EE + *B. breve* CCFM1025 groups (tested by one-way ANOVA, *P* < 0.05; **Supplementary Figure 1B** and **Supplementary Table 1**). Further, we performed multivariate analysis to identify discriminatory features between model and EE + *B. breve* CCFM1025 groups. Results from both orthogonal partial least squares-discriminant analysis (OPLS-DA) and hierarchical cluster analysis indicated distinct clusters of metabolites in fecal samples (**Figures 6A, B**, and **Supplementary Table 2**). As compared to model group, 44 differential metabolites (38 up-regulated and 6 down-regulated) were identified in the EE + *B. breve* CCFM1025 group by volcano plot (**Figure 6C**, **Supplementary Figure 2** and **Supplementary Table 3**), thus contributing greatly to metabolome discrimination. Functionally, these differentially altered metabolites were enriched in three metabolic pathways in feces, predominantly relevant to tryptophan metabolism (**Figure 6D**). Notably, three tryptophan metabolites, including tryptophan, xanthurenic acid, and indole-3-acetic acid altered in model mice were restored towards control levels by EE+1025 treatment (**Figures 6E–H**). Moreover, widespread decreases in 5-hydroxyindole acetic acid in fecal samples were observed in EE + *B. breve* CCFM1025 group compared to model group.

**Figure 7:**
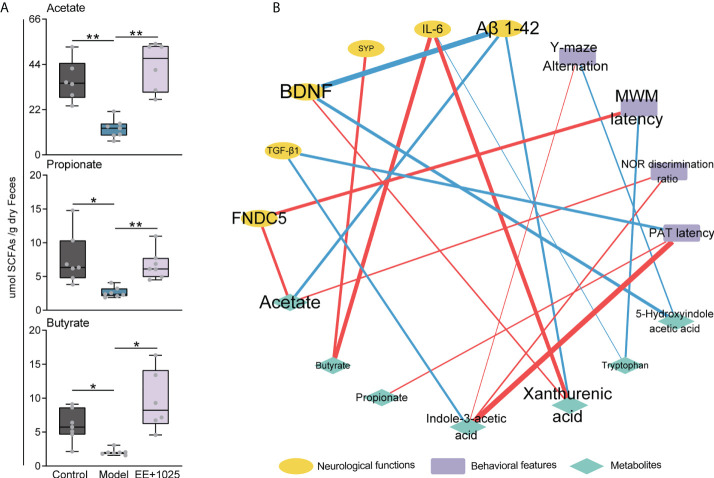
Integrated analysis reveals that brain function is associated with changes in microbial metabolites. **(A)** SCFA concentrations in feces. Data are presented as the median ± interquartile range. In the box plot, the bottom and top are, respectively, the 25th and 75th percentile, a line within the box marks the median. Whiskers above and below the box indicate 1.5 interquartile range of the lower and upper quartile, respectively. Each data point represents one independent mouse. *P < 0.05, **P < 0.01 as determined by one-way ANOVA. **(B)** Spearman correlation network containing behavioral features, neurological data and metabolites. Red edges indicate a positive correlation and blue edges indicate a negative correlation.

Given the role of SCFAs, which are also essential microbiota-derived metabolites, in modulating gastrointestinal physiology and neurological function, we further detected the concentrations of SCFAs in fecal samples using a GC–MS technique. We found that the levels of propionate, butyrate, and acetate were significantly decreased in the feces of model mice compared with control mice (**Figure 7A**). Notably, acetate, propionate, and butyrate levels were increased in the EE + *B. breve* CCFM1025 group, with acetate levels showing a greater increase than propionate and butyrate levels (**Figure 7A**). These results indicated that treatment with EE + *B. breve* CCFM1025 modulated the levels of microbiota-derived metabolites, some of which may contribute to the altered levels observed in the brain, and thus, confer neuroprotective effects against AD-associated brain deficits.

### Integrated analysis reveals that brain function is associated with changes in microbial metabolites

To identify putative microbiota–metabolite–brain interactions, we integrated behavioral and neurological data with metabolite levels using a correlation network. In this network, we found that specific metabolites differentially contributed to brain function (**Figure 7B**). Notably, the levels of Aβ_1-42_ were negatively correlated with BDNF, acetate, and xanthurenic acid levels. Moreover, we found that the MWM latency was positively correlated with FNDC5 levels (**Figure 7B**). This may be partially due to that the EE + *B. breve* CCFM1025 treatment induced higher BDNF and FNDC5 levels are beneficial to brain function. In addition, strong positive correlations were also observed between indole-3-acetic acid level and behavioral improvements (**Figure 7B**). These results were consistent with tryptophan metabolite being key regulator of neurotransmitters and crucial modulator of cognition.

## Discussion

In this study, we evaluated the effects of combined *B. breve* CCFM1025 and EE treatment on cognition, the composition of the gut microbiome, and microbial metabolite levels in AD mice. We found that cognitive impairment, neuroinflammation, and gut microbiota dysbiosis were key symptoms associated with the progression of AD. Compared with EE or *B. breve* CCFM1025 treatment alone, EE combined with *B. breve* CCFM1025 treatment showed a greater capacity to improve cognition and memory, reduce Aβ deposition, and inhibit neuroinflammation. Moreover, the findings from microbiome and metabolome analyses showed that EE combined with *B. breve* CCFM1025 treatment restored the structure of the gut microbiome, which was dysregulated in model mice, and altered microbial metabolite levels. Furthermore, metabolomic data integrated with behavioral and neurological data corroborated the microbiota–metabolite–brain interactions, with acetate and tryptophan metabolism as potential drivers. Given the numerous effects of EE and psychobiotics on cognition, behavior, synaptic plasticity, and microbial community structure, we propose that an intervention consisting of EE and *B. breve* CCFM1025 protects against cognitive deficits and slows the progression of AD, possibly by restructuring the gut microbiome and regulating acetate and tryptophan metabolism.

Amyloid accumulation is a core pathological hallmark of AD (1). In animal models of AD, intrahippocampal injection of Aβ_1-42_ mimics the Aβ-related neuropathological features of the AD brain, as its infusion can lead to behavioral alterations and cognitive deficits. Consistently, significant behavioral deficits, cognitive impairment, and synaptic damage were observed in Aβ-injected AD mice in this study. Recent studies have reported that psychobiotic interventions improve cognition and ameliorate depression in both animal and human studies (23). Additionally, previous studies have described the effect of exercise and probiotic treatment on cognition in different mouse models of AD (24–26). However, whether EE training alleviates AD-related pathology or probiotic administration has a neuroprotective effect have remained elusive. In this study, we confirmed that *B. breve* CCFM1025 treatment was sufficient to improve spatial reference memory, but its effect was not more significant than the effect of *B. breve* CCFM1025 + EE. However, *B. breve* CCFM1025 treatment was not sufficient to improve other forms of cognition. In contrast, EE combined with *B. breve* CCFM1025 treatment led to improved cognitive function in all four types of memory tests. The comprehensive improvements in cognition and memory may be due to the effect of EE, which provides the mice with more opportunities to perform a species-specific behavioral repertoire (14).

Previous studies have also confirmed the various positive effects of EE on the brain, including the promotion of adult hippocampal neurogenesis, improvements in learning and memory, and the enhancement of synaptic plasticity (13). Here, we found that EE-treated mice showed significantly decreased Aβ accumulation and highly elevated levels of BDNF, SYP, and FNDC5 compared with model mice housed in a standard cage, suggesting that exposure of mice to EE was critical for inhibiting neuroinflammation and increasing synaptic protein levels. Considering that these synaptic proteins are key modulators of synaptic plasticity, their increased levels may contribute to improved behavioral outcomes (27). Specifically, mice treated with EE + *B. breve* CCFM1025 showed increased levels of hippocampal BDNF, which is an essential neurotrophin that promotes many aspects of brain development and synaptic plasticity, whereas mice treated with *B. breve* CCFM1025 alone did not. This may be partially due to the fact that EE-induced exercise induces FNDC5, which stimulates the hippocampal expression of BDNF (28, 29). Similarly, only treatment of EE + *B. breve* CCFM1025 significantly increased the levels of hippocampal IL-6, another cytokine with selectively increased levels following exercise and which benefits cognition (27). As the components of EE include motor, sensory, cognitive, and social stimulation, no single factor explains the complexity underlying the multivariate effects of EE on the brain (13). Despite findings pointing to the effect of EE on cognition and synaptic plasticity, a more integrative view from different mechanistic perspectives is required.

It has become increasingly apparent that the gut microbiota is closely associated with neurological diseases through gut–brain connections, which may be regulated by neuronal and immune-mediated signaling (6). Moreover, accumulating evidence from both animal and clinical studies has revealed that gut microbiota dysbiosis plays a critical role in the progression of AD (2). Here, we found that Aβ injection markedly altered the structure of the gut microbiota. *Bifidobacterium* spp. is commonly considered as probiotic species. EE + *B. breve* CCFM1025 administration significantly increased the relative abundance of *B. longum*, and decreased the relative abundance of *B. pseudocatenulatum*. These alterations are similar to those associated with healthy brain function in normal individuals (30). Notably, *Bifidobacterium* was shown to be positively linked to behavior and cognition in mice. Consistently, previous studies have reported that the relative abundance of *B. adolescentis* is significantly decreased in mice with AD (17). In addition, one recently published study concluded that *Akkermansia muciniphila* and *Bilophila wadsworthia* abundance are strongly linked with cognitive impairment in patients with PD (31). Moreover, mice in the *B. breve* CCFM1025 group showed a marked increase in the abundance of *B. breve*, which may be due to the colonization of *B. breve* induced by its administration by oral gavage (32). Taken together, these results suggested that AD-related gut microbiota dysbiosis was recovered upon *B. breve* CCFM1025 treatment, which was critically associated with gut colonization by *B. breve* and in turn, stability of the gut microbiome.

One potential mechanism by which the gut microbiome may affect brain function and host health is *via* the microbes’ production of various metabolites, which convey signals from the gut to whole organs, including the brain (6). The most extensively studied microbiota-derived metabolites involved in the regulation of neurodegenerative diseases are amino acids and SCFAs (6). Amino acids are precursors for the biosynthesis of important neurochemicals and neurotransmitters, which can further affect the function of the central nervous system. A previous cohort study reported that people with AD have impaired amino acid metabolism (33) Similarly, functional prediction of gut microbiome genes revealed that metabolic outputs may be regulated by *B. breve* CCFM1025 alone or in combination with EE, predominantly related to amino acid metabolism. Furthermore, untargeted metabolomic profiles from fecal samples identified an important role of tryptophan metabolism. As one of the extensively studied amino acids, tryptophan can be metabolized by the gut microbiota. One recent study reported that hippocampal 5-hydroxyindole acetic acid levels increase with age (9). Here, we found that increased 5-hydroxyindole acetic acid levels were markedly restored by EE + *B. breve* CCFM1025 treatment, and were closely linked with improved cognition. In addition to tryptophan metabolites, SCFAs generated by the microbial fermentation of indigestible foods have also been reported to mediate gut–brain interactions to affect brain function. One recent study demonstrated that microbiota-derived acetate modulates the microglial phagocytosis of Aβ and disease progression in a mouse model of AD (34). Moreover, SCFA supplementation rescues cognitive and synaptic impairments, and alleviates microglial maturation defects. Here, the EE + *B. breve* CCFM1025-treated group showed a significantly higher concentration of acetate, which was suppressed in model mice. Numerous microbiota-derived metabolites are able to cross the BBB and thus, enter the brain and affect brain function (8). As such, EE + *B. breve* CCFM1025 treatment conferred neuroprotective effects against AD-associated brain deficits by modulating microbiota-derived metabolites, potentially driven by effects on acetate and amino acid metabolism.

There are some limitations of this study that should be addressed. First, we only demonstrated that EE training combined with *B. breve* CCFM1025 intervention provided a cognitive benefit to mice with AD. Whether these lifestyle intervention findings translate to patients with AD needs to be determined. Second, we primarily focused on the metabolic outcomes of EE combined with *B. breve* CCFM1025, but we are aware that, as an environmental factor, EE alone is critical for the progression of AD. Third, although we identified potential mechanisms from integrated data and correlation analyses, these experimental findings are not proof of causation. Finally, despite our increased awareness of the contribution of the gut microbiome and microbial metabolites to brain function, the mechanistic links and signaling molecules remain to be elucidated.

In conclusion, we demonstrated that EE training combined with *B. breve* CCFM1025 intervention attenuated AD-associated cognitive impairments and neuroinflammation. Furthermore, we found that the potential mechanism underlying these effects may involve the modulation of the gut microbiome and the regulation of acetate and tryptophan metabolism through gut–brain interactions. Although it is challenging to translate these findings to daily guidelines or clinical therapies, we provide an attractive multidomain intervention approach, with a combination of lifestyle-targeted and dietary microbiome-based interventions, to promote brain function and delay the progression of AD.

## Data availability statement

The data presented in the study are deposited in the National Center for Biotechnology Information repository, accession number PRJNA847039 (https://www.ncbi.nlm.nih.gov/bioproject/PRJNA847039).

## Ethics statement

The animal study was reviewed and approved by the Animal Experimentation Ethics Committee of Jiangnan University.

## Author contributions

Conceptualization, GZ and GW. data curation, JZ and WC. formal analysis: GZ. funding acquisition, GW and WC. investigation, GZ. methodology, HZ and MG. project administration, GW. resources, JZ and WC. software, MG. supervision, JZ and GW. validation, GW. visualization, GZ. writing – original draft, GZ. writing – review and editing, GW and WC. All authors contributed to the article and approved the submitted version.

## Funding

This work was supported by the National Natural Science Foundation of China (No. 31972052, 32021005, 31820103010), the Fundamental Research Funds for the Central Universities (JUSRP22006, JUSRP51501).

## Acknowledgments

Thanks for the help of the Collaborative Innovation Center of Food Safety and Quality Control in Jiangsu Province.

## Conflict of interest

The authors declare that the research was conducted in the absence of any commercial or financial relationships that could be construed as a potential conflict of interest.

## Publisher’s note

All claims expressed in this article are solely those of the authors and do not necessarily represent those of their affiliated organizations, or those of the publisher, the editors and the reviewers. Any product that may be evaluated in this article, or claim that may be made by its manufacturer, is not guaranteed or endorsed by the publisher.
