# Supplementary material for: Bifidobacterium breve intervention combined with environmental enrichment alleviates cognitive impairment by regulating the gut microbiota and microbial metabolites in Alzheimer’s disease mice
Source: Front Immunol. 2022 Sep 20;13:1013664. doi: 10.3389/fimmu.2022.1013664 (PMC9530393; doi:10.3389/fimmu.2022.1013664)
Supplement: Supplementary file 1 [file DataSheet_1.pdf]

Supplementary Material

1 Supplementary Methods

1.1 Behavioral tests

1.1.1 Y-maze

The Y-maze used in this study do not involve any training, reward, or punishment. Before testing, mice were habituated to the room for 30 min. Then, each mouse was placed in the center of the symmetrical Y-maze and was allowed to explore freely for 6 minutes. An alternation was considered by consecutive entry into all three arms. The number of maximum alternation was therefore the total number of arm entries minus 2, and the percentage of alternation was calculated as (actual alternations / maximum alternations) × 100.

1.1.2 Morris water maze

In MWM, mice were trained over five consecutive days during the acquisition phase, with four training trials using four start locations each day. On day six, the platform was removed and a probe trial lasting 60 s was conducted. The time to find the platform (escape latency) was the parameter to evaluate memory. Swimming trajectories were recorded using the Ethovision 11.5 automated tracking system (Noldus, Wageningen, Netherlands).

1.1.3 Novel object recognition task

Before testing, mice were habituated to the room for 60 min. On the pre-trial day, mice were placed in an empty, open arena and allowed to explore freely for 8 min. On the following day, each mouse was exposed for 8 min to two identical objects placed in the center of the arena (acquisition phase). 3 h later, one of the familiar objects was substituted with a novel object and the animal was allowed to explore the objects for 8 min (retention phase). Discrimination ratio was calculated according to the formula: (t(novel)−t(familiar)) / (t(novel)+t(familiar)).

1.1.4 Passive avoidance test

The apparatus chamber used in this test is composed of a black poorly illuminated compartment and a white illuminated compartment. Briefly, during the training test, the mouse was placed in the white compartment. When the mouse innately crossed to the black compartment, it received a mild foot shock. During the retention test, passive avoidance response was evaluated 24 h after foot-shock. Latency time was measured until the mouse entered the dark chamber completely.

1.2 Metabolomics

1.2.1 Metabolite sample preparation

Metabolites in fecal samples were extracted as detailed in Mars *et al* (1). Briefly, fecal samples were weighed on an analytical balance (sample weights ~50 mg) after which 1 mL of pre-cooled extraction solvent (methanol: acetonitrile: ultra-pure water = 2:2:1, v/v) were added to each sample, and vigorously mixed for 30 s. The fecal samples were then grinded by a frozen tissue grinder (60 Hz, 60 s) and extracted in a sonication bath for 20 min. Extracts were cleared of debris via centrifugation at

14,000 × g, for 15 minutes at 4 °C, and the resulting supernatant was transferred into a new microfuge tube. Samples were concentrated using a SpeedVac system (Thermo Fisher Scientific, MA, USA) and vacuum dried. Before analysis, dried samples were reconstituted in 50% acetonitrile (v/v) and transferred to a glass autosampler vial. For quality control purpose, a QC sample was prepared by pooling 10 uL of every sample.

1.2.2 HPLC-MS analysis parameters

The metabolite samples were analyzed with an UItiMate 3000 HPLC system (Thermo Fisher Scientific, MA, USA) coupled to a high-resolution Q Exactive mass spectrometer (Thermo Fisher Scientific, MA, USA). Chromatographic separation was achieved on a Waters Acquity UPLC HSS T3 column (2.1 × 100 mm, 1.8 µm; Waters, Milford, USA) over a 15 min gradient. Mobile phases were A) 0.1% formic acid in postive mode or 5 mM ammonium acetate in negative mode and B) 100% acetonitrile. The analytical gradient was: 0-1 min, 2% B; 1-10 min, 98% B; 10-12 min, 98% B; 12.1 min, 2% B; 12.1-15 min, 2% B. Flow rate was 0.3 mL/min with an injection volume of 2 uL for both. Samples were held at 4°C in the autosampler, and the column was operated at 35°C.

ESI source parameters were set as follows: spray voltage, 3.5 KV in positive or negative modes, respectively; sheath gas, 35 arb; aux gas, 15 arb; aux gas heater temperature, 320 °C; capillary temperature, 320 °C. The Q Exactive was run with polarity switching in full scan ddMS2 mode for all samples and QC samples to acquire MS/MS spectra. The full scan parameters were set as: resolution, 70,000; AGC target, 1e6; maximum injection time, 100 ms; scan range, 70–1050 m/z. The ddMS2 scan parameters were set as: resolution, 17,500; AGC target, 1e5; maximum injection time, 50 ms; scan range, 70–1050 m/z; top N setting, 10; isolation width, 1.5 m/z; collision energy mode, stepped; collision energy type, normalized; normalized collision energy (NCE) at 20, 40, 60 eV; dynamic exclusion duration was set as 7 s for excluding after 1 time.

2 Supplementary Figures and Tables

2.1 Supplementary Figures

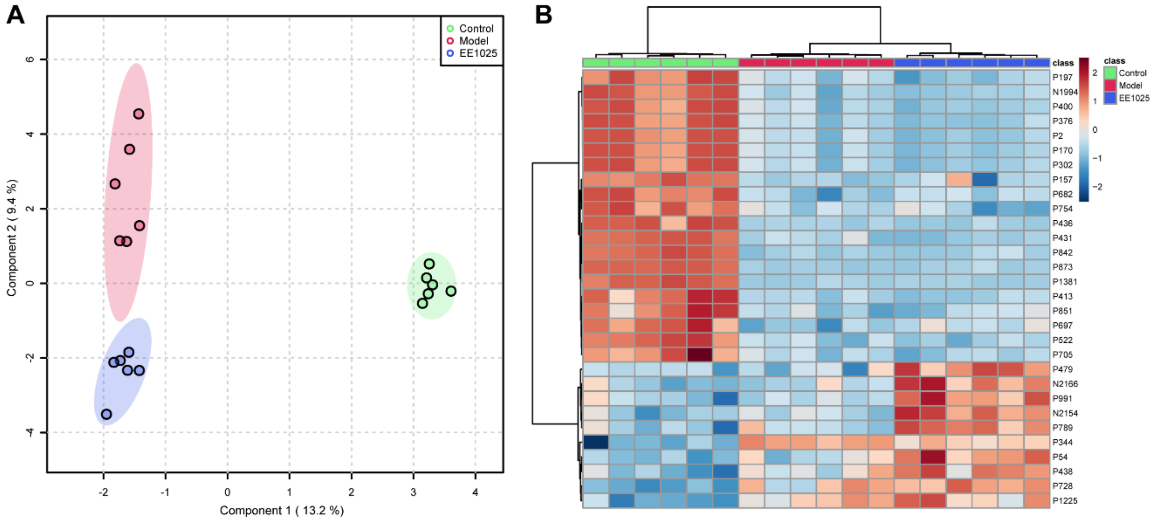

Supplementary Figure 1. Multivariate analysis of identified metabolites among three groups. (A) Sparse PLS-DA score plot for fecal samples among three groups. Number of components: 6. (B) Hierarchical cluster analysis of differential metabolites (top 30). The name of differential metabolites was listed in Supplementary Tables S1.

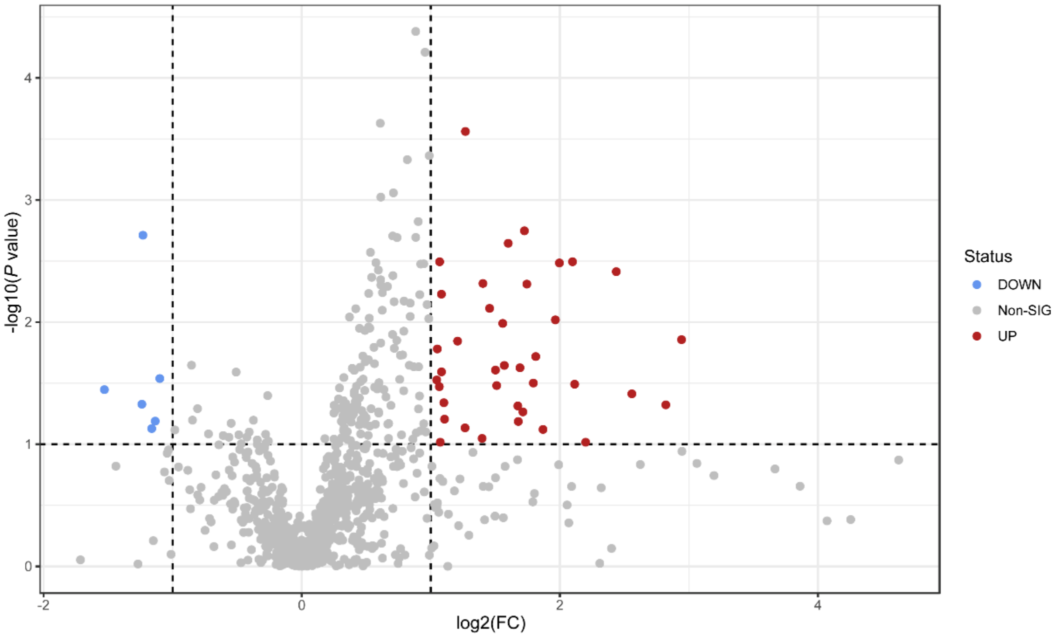

Supplementary Figure 2. Important features selected by volcano plot between model and EE + *B. breve* CCFM1025 groups. Fold change > 2, adjusted *P* value < 0.1. Metabolites with significant changes are presented in red (upregulated) or blue (downregulated).

2.2 Supplementary Tables

Supplementary Table 1. The differential metabolites in feces among three groups using one-way ANOVA.

Metab_ID	Metabolite	P value	FDR
P873	Dihydrozeatin	6.92E-17	6.40E-14
P1381	Praeruptorin	1.33E-15	6.14E-13
P842	1-Propyl-1H-purin-6-amine	4.43E-13	1.37E-10
P431	N,N-Bis(2-hydroxyethyl)dodecanamide	1.94E-11	4.48E-09
P436	Alvameline	1.34E-10	2.48E-08
P2	Bis(4-ethylbenzylidene) sorbitol	1.13E-09	1.74E-07
N1994	TU4153400	2.11E-09	2.79E-07
P376	2,4-Dimethylbenzaldehyde	2.99E-09	3.19E-07
P170	Piribedil	3.10E-09	3.19E-07
P302	8'-Hydroxyabscisate	5.33E-09	4.93E-07
P522	Nonyltrimethylammonium	8.15E-09	6.85E-07
P400	1_5-Anhydro-D-mannitol	9.08E-09	7.00E-07
P682	12,13-Epoxytrichothec-9-ene-3,4,8,15-tetrol	1.89E-08	1.34E-06
P197	alpha-methylstyrene	2.30E-08	1.52E-06
P413	1872050	1.37E-07	8.46E-06
P697	Cedefingol	2.61E-07	1.51E-05
P344	11Z_8Z_14Z-eicosatrienoicacid	7.53E-07	4.10E-05
P754	2,3,4,5,6-pentamethylphenyl	1.31E-06	6.76E-05
N2154	NP-005196	2.33E-06	0.000113
P728	(4E,7E,10E,13E,16E)-Docosapentaenoyloxy	5.68E-06	0.000263
P705	N,N-Dimethyldecylamine N-oxide	6.44E-06	0.000284
P851	N-Nonanoylglycine	1.27E-05	0.000536
P157	Triethanolamine	1.35E-05	0.000541
P54	I-Urobilinogen	1.49E-05	0.000575
N2166	Protectin D1	2.43E-05	0.000899
P479	N-(3,5-Dimethoxybenzoyl)glycine	3.52E-05	0.001251
P789	Dihydrophaseicacid	3.82E-05	0.00131
P991	5-hydroxy-3,4-dihydro-2H-naphthalen-1-one	3.98E-05	0.001315
P1225	N-Vanillyloleamide	5.35E-05	0.001707
P438	tilisolol	8.63E-05	0.002662

Supplementary Table 2. The differential metabolites in feces between model and EE + *B. breve* CCFM1025 groups using T-test.

Metab_ID	Metabolite	P value	FDR
N2154	NP-005196	4.17E-05	0.028522
P991	5-hydroxy-3,4-dihydro-2H-naphthalen-1-one	6.17E-05	0.028522
P789	Dihydrophaseicacid	0.000235	0.063489
N2166	Protectin D1	0.000275	0.063489
P479	N-(3,5-Dimethoxybenzoyl)glycine	0.000433	0.072052
P1295	3-Methyl-1-(2_4_6-trihydroxyphenyl)butan-1-one	0.000467	0.072052
P409	Didecyldimethylammonium	0.000874	0.10944

P69	2-Hydroxycinnamic acid	0.000946	0.10944
P1339	(DL)-3-O-Methyldopa	0.001501	0.13404
P54	I-Urobilinogen	0.001787	0.13404
P363	N1_N12-Diacetylspermine	0.001941	0.13404
P223	dihomomethionine	0.001969	0.13404
P428	Pilocarpine	0.002023	0.13404
P1524	4-methoxy-6-(prop-2-en-1-yl)-2H-1,3-benzodioxole	0.002029	0.13404
N2305	3_4-Dimethoxycinnamicacid	0.002262	0.13946
P664	2,6-DIAMINO-4-NITROTOLUENE	0.002685	0.14068
N2144	prohydrojasmon	0.003203	0.14068
N2292	1,7-Dihydroxy-6,6-dimethyl-3,5,5a,6,7,8,9a,9b-octahydronaphtho[1,2-c]furan-9(1H)-one	0.003205	0.14068
P356	L-Tyrosinemethylester	0.003258	0.14068
P1652	Civetone	0.003275	0.14068
N2167	Alachlor ESA	0.003333	0.14068
P158	3-(propan-2-yl)-octahydropyrrolo[1,2-a]pyrazine-1,4-dione	0.003346	0.14068
P277	IRH	0.003749	0.1488
N2246	NP-004768	0.003861	0.1488
P420	asn-pro	0.00416	0.15222
P1194	Trilostane	0.004299	0.15222
P1051	NP-018711	0.004496	0.15222
P856	6-Benzamidopurine	0.004824	0.15222
P1594	Pregnenolone	0.00488	0.15222
N1950	5-Hydroxy-3-(4-hydroxy-3-methoxyphenyl)-7-methoxy-8-methyl-2,3-dihydro-4H-chromen-4-one	0.004964	0.15222

Supplementary Table 3. The significantly altered metabolites used for pathway enrichment analysis.

Metab_ID	Metabolite	P value	HMDB ID	KEGG ID
P54	I-Urobilinogen	0.001787	HMDB0001898	C05790
P138	N8-Acetylspermidine	0.028946	HMDB0002189	C01029
P144	5-Hydroxyindoleacetic acid	0.033765	HMDB0000763	C05635
P156	tranexamic acid	0.025543	HMDB0014447	C12535
P167	Xanthurenic acid	0.065116	HMDB0000881	C02470
P173	(9S_10S)-9_10-Dihydroxyoctadecanoate	0.035708	HMDB0059633	C15988
P205	7-Methylguanine	0.062308	HMDB0000897	C02242
P212	Prolylleucine	0.054318	HMDB0028867	-
P262	Adenosine	0.005903	HMDB0000050	C00212
P266	3-Aminosalicylic acid	0.038646	HMDB0001972	-
P275	gamma-Glutamyl-gamma-aminobutyraldehyde	0.010239	METPA1197	C15700
P329	Tryptophan	0.032236	HMDB0000929	C00078
P331	Indole-3-acetic acid	0.096269	HMDB0000197	C00954
P337	Xanthurenic acid	0.024687	HMDB0000881	C02470
P338	Geranylacetate	0.04758	HMDB0035157	C09861
P363	N1_N12-Diacetylspermine	0.001941	HMDB0002172	C03413

P379	Kynurenic acid	0.033077	HMDB0000715	C01717
P415	4-Octyl-1,3-cyclopentanedione	0.013916	-	-
P422	(1S,2R,5S)-2-Isopropyl-5-methylcyclohexyl 3-oxobutanoate	0.019127	-	-
P468	N2-(D-1-Carboxyethyl)-L-lysine	0.047	METPA0456	C04020
P496	N-(3,5-Dimethoxybenzoyl)glycine	0.073422	HMDB0029083	-
P499	Pseudouridine	0.096263	HMDB0000767	C02067
P575	asp-leu	0.075649	HMDB0028757	-
P606	NP-018660	0.023641	-	-
P622	NP-019811	0.045701	-	-
P707	N,N-Dimethyl-N-phenylurea	0.029772	HMDB0001020	C02846
P749	3082	0.048546	HMDB0003424	C00823
P810	Isorhapontigenin	0.031545	HMDB0128522	-
P856	6-Benzamidopurine	0.004824	-	-
P1053	DA9185000	0.022621	-	-
P1054	(2R)-2-{[(1R,2S,3R,4R,5R)-4-Acetamido-2-hydroxy-6,8-dioxabicyclo[3.2.1]oct-3-yl]oxy}propanoic acid	0.064682	-	-
P1090	10-HDA	0.014298	HMDB0060037	-
P1594	Pregnenolone	0.00488	HMDB0000253	C01953
P1652	Civetone	0.003275	HMDB0031336	-
N1935	Nonanoic acid	0.074426	HMDB0000847	C01601
N2011	Rhapontigenin	0.089517	HMDB0031842	-
N2067	5-Nonyl-2-oxotetrahydro-3-furancarboxylic acid	0.007709	-	-
N2113	NP-021701	0.00958	-	-
N2144	prohydrojasmon	0.003203	-	-
N2149	(2S)-N2-[(2S)-1-amino-3-cyclohexyl-1-oxo-2-propanyl]-N1-isopropyl-N4-phenyl-1,2,4-piperazinetricarboxamide	0.016578	-	-
N2166	Protectin D1	0.000275	HMDB0003689	-
N2246	NP-004768	0.003861	-	-
N2292	1,7-Dihydroxy-6,6-dimethyl-3,5,5a,6,7,8,9a,9b-octahydronaphtho[1,2-c]furan-9(1H)-one	0.003205	-	-
N2305	3_4-Dimethoxycinnamicacid	0.002262	HMDB0032226	-
